# Translation into Portuguese (Brazil), cultural adaptation and validation of Parathyroid Assessment of Symptoms (PAS) in patients with chronic kidney disease and hyperparathyroidism

**DOI:** 10.1590/2175-8239-JBN-2022-0059en

**Published:** 2024-10-14

**Authors:** Rafael Costa e Campos, Rayssa Ruszkowski do Amaral, Marta Ribeiro Hentschke, Marcelo Garcia Toneto, Carlos Eduardo Poli-de-Figueiredo

**Affiliations:** 1 Pontifícia Universidade Católica do Rio Grande do Sul, Escola de Medicina, Porto Alegre, RS, Brazil.

**Keywords:** Hyperparathyroidism, Quality of Life, Renal Insufficiency, Chronic, Reproducibility of Results, Validation Study

## Abstract

**Introduction:**

Chronic kidney disease (CKD) is related to high morbidity and mortality and loss of quality of life. Likewise, hyperparathyroidism is associated to progressive loss of renal function, with increased phosphate and decrease calcium levels, which induce the secretion of parathyroid hormone.

**Objectives:**

To translate into Portuguese (Brazil), culturally adapt and validate the questionnaire Parathyroid Symptoms Assessment (PAS), following reliability and validity criteria in patients with chronic kidney disease and hyperparathyroidism.

**Methods:**

Methodological and cross-sectional study, carried out at São Lucas Hospital/PUCRS, Porto Alegre, Brazil. The PAS questionnaire validation process followed protocols from previous studies. After translating into Portuguese, it was applied to 100 patients with secondary (SHPT) and tertiary or persistent (THPT) hyperparathyroidism. For PAS validation data, patients responded to the Short Form Health 36 (SF-36) questionnaire. Reliability criteria were evaluated using intraclass correlation coefficient (ICC) and Cronbach’s alpha (α-C). Validity was assessed by Spearman’s correlation coefficient between PAS and SF-36 values.

**Results:**

Participant’s mean age was 55.6 ± 15.6 years, 61% was male, and 68% was diagnosed with SHPT. Among 100 patients, 53% performed a PAS retest (ICC = 0.83). The internal reliability by α-C was 0.86. Negative correlations were observed between PAS questions and SF-36 physical and mental domains, which ranged from 0.3 to 0.7.

**Conclusion:**

The Brazilian version of the PAS questionnaire was found to be valid and reliable. The PAS questionnaire can be used to evaluate quality of life in Brazilian patients with hyperparathyroidism who speak Portuguese.

## Introduction

It is estimated that chronic kidney disease (CKD) can affect 8 to 16% of the world’s population. The main causes of CKD are diseases with high prevalence and incidence, such as diabetes mellitus (DM) and arterial hypertension (AH)^1^. People with stage 5 CKD (glomerular filtration rate (GFR) <15 mL/min/1.73m^2^) have end stage renal disease and require renal replacement therapy by dialysis or kidney transplantation. As renal failure progresses, usually with a GFR ≤ 60 mL/min/1.73m^2^, changes occur in serum concentrations of calcium, phosphate, vitamin D, and parathyroid hormone (PTH), which are associated with vascular and soft tissue calcifications, as well as alterations in bone remodeling. This systemic syndrome is called chronic kidney disease-mineral and bone disorder (CKD-MBD)^2^. Metabolic alterations such as hypocalcemia, hyperphosphatemia, and vitamin D deficiency stimulate increased PTH secretion, leading to the development of secondary hyperparathyroidism (SHPT).

In Brazil, around 50% of dialysis patients have SHPT and usually present significant symptoms such as bone pain, joint pain, myalgia, muscle weakness, itching, irritability, among others, which reduce their quality of life^3^. With the progression of secondary hyperparathyroidism (SHPT), in addition to the mentioned complications, patients experience a reduction in bone mineral density and alterations in bone microarchitecture, which increase the risk of pathological fractures and mortality. Patients with severe SHPT that does not respond to clinical treatment are referred for parathyroidectomy (PTx).

The evaluation of signs, symptoms, and quality of life through scales or questionnaires has attracted the interest of health professionals, leading to numerous publications in recent years. These instruments aim to assess the physical, mental, and social aspects of the patient and express them as quantitative variables.

The “Parathyroid Assessment of Symptoms” (PAS) questionnaire is a tool developed by Pasieka and Parsons^4^, which assesses specific symptoms of hyperparathyroidism. Initially, the questionnaire was applied only to patients with primary hyperparathyroidism (PHPT), both pre- and post-parathyroidectomy (PTx). Later, it was also validated for secondary hyperparathyroidism (SHPT) and persistent or tertiary hyperparathyroidism (THPT), which occurs after kidney transplantation. This questionnaire is used to confirm the diagnosis of the disease, assess the severity of symptoms, and compare its values before and after PTx, evaluating the impact of surgical success on these symptoms. Previously, generic instruments such as Short Form Health 36 (SF-36) were used to measure quality of life in patients with hyperparathyroidism^5^.

Thus, due to the clinical importance of PAS, this study aimed to translate, culturally adapt, and validate into Portuguese (Brazil) the PAS questionnaire, following reliability and validity criteria in patients with CKD and hyperparathyroidism.

## Methods

### Translation and Cultural Adaptation

Initially, the researcher Pasieka JL, responsible for the development of the original PAS questionnaire, was contacted to obtain permission to translate the PAS into Portuguese^6^. The translation and cultural adaptation followed guidelines established in the literature^7,8^. The process started with the translation of the PAS from the original language (English) into the target language (Portuguese) by two independent translators whose native language was Portuguese. The two translations were analyzed, and a first version was produced. Afterwards, a back translation was performed where the first Portuguese version was back-translated into the original language by a third professional translator whose native language was also Portuguese. The second version (English) was analyzed and compared with the first version (Portuguese) by five experts in the area and by the author of the original questionnaire. For cultural adaptation, the analysis was carried out by a group of experts in the field selected by the authors. The pre-test was applied in five patients: two with PHPT, two with SHPT, and one with THPT^9^.

### Study Design and Sample

The study was characterized as methodological in the translation and cultural adaptation phase and as transversal in the validation phase. For the validation phase, 100 patients were included, both men and women, aged 18 years or older, diagnosed with CKD and SHPT or renal transplant patients with THPT, who underwent follow-up and treatment at Nephrology and Dialysis Unit (São Lucas Hospital (HSL)/PUCRS, Porto Alegre, Brazil). All patients were fluent in Portuguese. Both questionnaires were applied after obtaining a signed informed written consent from the participant.

The study was approved by the institutional scientific and ethics research committee (No. 2.292.273-CEP).

#### Operational definitions of SHPT and THPT

SHPT: Presence of elevated PTH (reference value (RV) 15 to 68.3 pg/mL), normal or low calcium (total calcium RV 8.4 to 10.2 mg/dL) in patients with stage 5 CKD.

THPT: Presence of elevated PTH, normal or high calcium in kidney transplant patients.

The RV used were those recommended by the hospital where the study was performed.

#### Exclusion criteria

Patients with physical limitations that affected the ability to answer the questionnaire and patients whose treatment was changed or who underwent surgery (parathyroidectomy and kidney transplantation) during the time between questionnaire applications.

#### Sample calculation

Sample calculation followed the guideline of 5 to 10 participants per questionnaire item (the PAS questionnaire has 13 items)^10^.

### Demographic, Clinical, and Biochemical Data

Patients’ demographic (sex, ethnicity), clinical (age, weight, height, type and time of dialysis, time since renal transplantation), and laboratory (PTH, creatinine, serum calcium and alkaline phosphatase) variables were retrieved from HSL records.

### Measuring Instruments

#### Quality of life assessment

#### Parathyroid assessment of symptoms

The PAS is a specific questionnaire for patients with hyperparathyroidism consisting of 13 questions (physical, mental, and social). Each item is scored according to the response on a visual analog scale ranging from zero (no symptoms) to 100 (extreme symptoms). The PAS score is calculated as the sum of all 13 answers, with a maximum score of 1300.

#### SF-36, short form health 36

The SF-36 is a generic quality of life instrument consisting of 36 questions, where the patient responds to varying questions with scores between 1 or 2, 1 to 3, 1 to 5, and 1 to 6. The 36 items are divided into 8 domains: functional capacity, physical aspects, pain, general health, vitality, social aspects, emotional aspects, and mental health. Each domain generates a score between zero (worst state) and 100 (best state). The SF-36 instrument was applied in the study population as the gold standard, so that construct criterion validity could be assessed.

The PAS and SF-36 questionnaires were applied by the same person to all patients.

### Reliability and Validity

The instrument reliability was assessed using reproducibility by intraclass correlation (test/retest)^11,12^ and internal consistency by Cronbach’s alpha (α-C)^13^. By calculating the intraclass correlation coefficient (ICC) between two applications (test/retest), the ability of the instrument to provide similar results in a given time period was assessed. Convergent validity was performed by assessing the correlation between the results obtained in PAS and SF-36.

### Statistical Tests

Statistical tests were conducted using the RStudio program (version 1.0.143–2009–2016 RStudio^©^, Inc.). Quantitative variables are presented as mean ± standard deviation (SD) or median and interquartile range (IQR) as appropriate. Categorical variables are presented in percentages. PAS questions and SF-36 domains are reported as median and IQR, in addition to minimum and maximum values.

For the analysis of test/retest reliability, the ICC was used, with values between 0 and 1. According to the literature, ICC values <0.5 indicate low reliability, between 0.5 and 0.75 moderate reliability, 0.75 to 0.9 good reliability, and >0.9 excellent reliability^14^. For a good internal reliability, α-C coefficient should be >0.7^15,16^.

The correlation between PAS and SF-36 results was tested with Spearman’s correlation coefficient^17^. The null hypothesis was rejected when *p* < 0.05.

## Results

### Demographic, Clinical, and Biochemical Data

Between January and October 2018, a total of 100 patients with diagnosis of SPHPT (68%) and TPHPT (32%) were recruited for validation of the PAS in Portuguese (61 men; aged 21–81, mean 55.6 ± 15.1 years). The interviews were carried out during outpatient care. The mean body mass index (BMI) was 26.9 ± 6.0 kg/m^2^. According to the WHO, the majority of patients had overweight (BMI ≥ 25 < 30)^18^.

The underlying disease leading to CKD in patients with SHPT and THPT was DM in 22 patients, hypertension in 16, urological disease in 7, other renal disease in 32, and indeterminate causes in 23. Among other underlying renal diseases there were glomerulopathies in 11 patients, polycystic kidney disease in 8 patients, autoimmune diseases in 5, tumor and agenesis in two patients each, and pyelonephritis, alport, tuberculosis, and ischemic nephropathy in one patient each.

Among participants diagnosed with SPTH, 44 (64.7%) underwent hemodialysis as renal replacement therapy, 10 (14.7%) peritoneal dialysis, and 6 (8.82%) underwent both therapies, never simultaneously, prior to the study period. Eight patients (11.76%) had not yet started dialysis therapy. The median time from dialysis onset to interview day was 1.8 years [IQR 0.9–3.3]. Previous kidney transplantation was found in 17 (25%) patients, which later progressed to loss of graft function.

Among participants diagnosed with TPTH, the median post-transplant time to the interview day was 4.2 years [IQR 2.5–5.7]. Regarding type of donor, 31 were deceased donors (97.9%). The median time to dialysis, prior to transplantation, was 3.9 years [IQR 2–5.8]. Regarding renal replacement therapy, 21 (65.6%) were on hemodialysis, 4 (12.5%) on peritoneal dialysis, and 6 (18.7%) on both therapies, never simultaneously, prior to the study period. Among transplanted patients with THPT, 50% had SHPT before transplantation (during the dialysis period). Demographic and clinical characteristics are shown in Table 1.

**Table 1:** Demographic and clinical characteristics

Data (n = 100)	
Gender	
Male	61%
Age (years)	55.6 (±15.1)^1^
Weight (kilograms)	74.87 (±16.7)^1^
Height (meters)	1.67 (±0.1)^1^
BMI (kg/m^2^)	26.94 (±6.0)^1^
Skin color	
White	50%
Non-white	42%
No answer	8%
Hyperparathyroidism	
Secondary	68%
Tertiary	32%
Time from dialysis onset to interview (years)	1.8 [0.9–3.3]^2^
Post-transplant time to the interview (years)	4.2 [2.5–5.7]^2^

BMI, body mass index.

1 Mean (±standard deviation);

2 Median [interquartile range].

The biochemical test results, including glomerular filtration rate (GFR, using concepts of “*The Chronic Kidney Disease Epidemiology Collaboration*” (CKD-EPI) equation)^19^, are shown in Table 2.

**Table 2:** Laboratory tests and glomerular filtration rate (GFR)

Variable	Median [IQR]
Creatinine (mg/mL)	
SHPT	8.8 [6.23–11.45]
THPT	1.79 [1.55–3.02]
SHPT + THPT	6.29 [3.23–9.57]
Estimated GFR (mL/min/1,73m^2^)	
SHPT	5.55 [4.38–6.48]
THPT	44.3 [19.57–55.3]
SHPT + THPT	7.9 [4.87–17.03]
PTH (pg/mL)	
SHPT	504.3 [274.4–884]
THPT	242.25 [143.8–339.1]
SHPT + THPT	425 [233.7–733.2]
Serum calcium (mg/dL)	
SHPT	8.95 [8.58–9.5]
THPT	10.1 [9.3–11.03]
SHPT + THPT	9.2 [8.7–9.83]
Alkaline phosphatase (U/L)	
SHPT	124 [85–164]
THPT	114 [83.5–195]
SHPT + THPT	123 [85–165.5]

PTH: Parathyroid hormone; [IQR]: interquartile range; SHPT: Secondary hyperparathyroidism; THP: Tertiary hyperparathyroidism.

### Measuring Instruments

The PAS and SF-36 questionnaires were applied in 100 patients. The second application of PAS was performed in 53 of those patients, with a median of 16 days between both applications. Of the 53 patients, 45 (84.1%) had SHPT and 8 (15.1%) had THPT. No secondary application of SF-36 was made.

The ICC between the two applications (test/retest) was 0.83. When including only applications with ≤16 days interval (32 patients), the ICC was 0.84. The α-C for internal reliability was 0.86 in the whole sample (SHPT= 0.86 and THPT= 0.88). The minimum value was 0.82 and maximum was 0.9 for the 13 PAS questions. Reliability and PAS data are shown in Table 3.

**Table 3:** Parathyroid assessment symptoms (PAS) values and reliability tests

Variable	Median [IQR]
PAS	
SHPT	340 [237.5–512.5]
THPT	290 [230–515]
SHPT + THPT	332.5 [230–512.5]
Interval between PAS applications (days)	16 [14–21]
Intraclass correlation coefficient^1^	0.83
Cronbach’s Alpha	
SHPT	0.86
THPT	0.88
SHPT + THPT	0.86

[IQR]: interquartile range.

PAS values range from 0 to 1300.

1 Calculated in 53 patients who performed the second interview of the PAS.

SHPT: Secondary hyperparathyroidism; THPT: Tertiary hyperparathyroidism.

PAS item values were (range from 0 to 100): Pain in the bones 10 [0–42.5]; Feeling tired easily 35 [8.75–72.50]; Mood swings 30 [0–50]; Feeling depressed 30 [0–60]; Pain in the abdomen 0 [0–30]; Feeling weak 30 [10–50]; Feeling irritable 30 [0–50]; Pain in the joints 30 [0–60]; Forgetfulness 30 [8.75–50]; Difficulty getting out of a chair or car 10 [0–40]; Headaches 0 [0–30]; Itchy skin 17.5 [0–50]; Being thirsty 45 [20–72.5].

The SF-36 scale values were (range 0 to 100): Physical functioning (10 items) 55 [35–81.25]; Role-physical (7 items) 50 [0–100]; Bodily pain (2 items) 61 [41–74]; General health (5 items) 47 [35–67]; Vitality (4 items) 60 [43.75–70]; Social functioning (1 item) 75 [50–100]; Role-emotional (5 items) 66.67 [0–100]; and Mental health (1 item) 68 [52–84].

The correlations between the first application of PAS and SF-36 are described in Table 4. Spearman’s correlation values and their significance (*p*) are shown.

**Table 4:** Spearman’s correlation between the results of the parathyroid assessment symptoms (PAS) and the short form health 36 (SF-36)

	*SF-36*							

PAS	PF	RP	Pain	GH	VT	SF	RE	MH
*Pain in the bones*	−0.33^1^	−0.33^1^	−0.47^1^	−0.23^2^	−0.35^1^	−0.32^2^	−0.08	−0.30^2^
*Feeling tired easily*	−0.47^1^	−0.28^2^	−0.33^1^	−0.30^2^	**−0.51^1^**	−0.36^1^	−0.11	−0.41^1^
*Mood swings*	−0.15	−0.14	−0.19	−0.30^2^	−0.39^1^	−0.39^1^	−0.25^2^	**−0.61^1^**
*Feeling depressed*	−0.23^2^	−0.13	−0.14	−0.36^1^	−0.34^1^	−0.28^2^	−0.29^2^	**−0.65^1^**
*Pain in the abdomen*	−0.14	−0.15	−0.39^1^	−0.02	−0.13	−0.19	0.04	−0.18
*Feeling weak*	−0.44^1^	−0.33^1^	−0.36^1^	−0.39^1^	**−0.62^1^**	−0.40^1^	−0.19	−0.41^1^
*Feeling irritable*	−0.12	−0.01	−0.14	−0.19^2^	−0.32^2^	−0.26^2^	−0.14	−0.48^1^
*Pain in the joints*	−0.36^1^	−0.31^1^	**−0.62^1^**	−0.29^2^	−0.27^2^	−0.33^1^	−0.11	−0.29^2^
*Forgetfulness*	−0.07	−0.06	−0.06	−0.06	−0.17	−0.17	−0.03	−0.23^2^
*Difficulty getting out of a chair or car*	**−0.61^1^**	**−0.50^1^**	−0.42^1^	−0.34^1^	**−0.51^1^**	**−0.52^1^**	−0.20^2^	−0.39^1^
*Headaches*	−0.13	−0.23^2^	−0.27^2^	−0.16	−0.30^2^	−0.25^2^	−0.18	−0.31^1^
*Itchy skin*	−0.34^1^	−0.41^1^	−0.37^1^	−0.41^1^	−0.49^1^	**−0.50^1^**	−0.17*	−0.39^1^
*Being thirsty*	−0.37^1^	−0.30^2^	−0.32^2^	−0.20^2^	−0.33^1^	−0.25^2^	−0.15	−0.21^2^

PF: Physical functioning; RP: Role-physical; GH, General health; VT: Vitality; SF: Social functioning; RE: Role-emotional; MH: Mental health.

Correlation magnitude. 0 to −0.1: trivial, −0.1 to −0.3: low, −0.3 to −0.5: moderate, −0.5 to −0.7: high, −0.7 to −0.9: very high, and −0.9 to −1: almost perfect.

**Featured values**: correlation ≤ −0.5^1^; p < 0.001^2^; p < 0.05.

## Discussion

In this study, the PAS questionnaire was translated from English (original instrument language) into Portuguese (Brazil), and the new version underwent cross-cultural adaptation (after expert analysis) and validation (by comparing its results with those of the SF-36 questionnaire).

The α-C obtained with the application of the PAS questionnaire to the study sample was high, proving the reliability of the new instrument. The ICC obtained by test/retest presented a favorable result, even when the interval between the two PAS applications was greater than 16 days, certifying the reliability of the instrument.

To test the reproducibility of an instrument through test/retest, the time between the first and second application should not be too short, so that the patient remembers the answers. On the other hand, the time between applications should not be too long either, so that the interviewer does not acquire new experiences that could affect the results. Ideally, the test/retest should be performed within a two-week period^10,11^.

In the literature review, we found at least three other studies that applied THE PAS in patients with SHPT^3,20,21^. Pasieka and Parsons^3^ assessed the impact of parathyroidectomy on symptoms in patients with SHPT and THPT and is in concordance with the present study.

Regarding the application of the SF-36 in patients with CKD and SHPT diagnosis, previous studies found a decrease in quality of life indicators mainly in physical health items^22^, which are in accordance with our study^21,23, 24, 25^.

PAS scores ranges from zero (no symptoms) to 100 (extreme symptoms) and SF-36 ranges from zero (worst state) to 100 (best state). Thus, when establishing correlations between PAS and SF-36 we obtained negative values, showing an inverse correlation between both.

Previous studies found significant correlations between PAS questions and SF-36 scores regarding physical and mental subgroups^20,26^. These studies concluded that PAS (13 questions) could be part of the medical routine as it assesses more specifically the symptoms and quality of life of patients with hyperparathyroidism.

In the present study, the correlations between both questionnaires were compatible with the content of each question. For example, physically related questions in the PAS were closely to the SF-36 physical domains, as were emotional wellbeing questions with mental domains.

Moderate and high correlations were seen in almost all analyses between the PAS and SF-36. These results confirm the consistency and concordance of both questionnaires.

The PAS and SF-36 results suggest that there is a loss in quality of life in patients with hyperparathyroidism, although this was not the main study objective.

This study had some limitations. First, it was a cross-sectional study with a heterogeneous sample, which led us to consider PTH values for the definition of SHPT based on laboratory-established standards. Nevertheless, the median PTH in SHPT was 504.3 [IQR 274–884]. Additionally, despite its relevance, serum phosphorus values were not presented due to a lack of data in the medical records. Also, we had difficulties in performing the retest within two weeks, as occurred in previous studies. Nevertheless, this does not seem to have influenced the ICC calculation. The only PAS question that was not correlated with any SF-36 domain was “Forgetfulness”, probably because there is no such item in the SF-36. Finally, none of the items of the PAS and SF-36 were very highly correlated (*p* ≥ −0.7). Most correlations were moderate or high, which seems to be sufficient for the validation of the instrument validation.

## Conclusion

The Portuguese version of the PAS (Brazil) showed good reliability and validity and was adapted to evaluate quality of life of Portuguese-speaking patients with hyperparathyroidism.

The clinical application of the present version of the PAS before and after an medical or surgical intervention, will provide information on the test’s ability to assist diagnoses, identify symptoms and evaluate improvements in quality of life after treatments.
