# Supplementary material for: Translation into Portuguese (Brazil), cultural adaptation and validation of Parathyroid Assessment of Symptoms (PAS) in patients with chronic kidney disease and hyperparathyroidism
Source: J Bras Nefrol. 2024 Oct 14;46(4):e20220059. doi: 10.1590/2175-8239-JBN-2022-0059en (PMC11475941; doi:10.1590/2175-8239-JBN-2022-0059en)
Supplement: Translation

# Translation: Tradução para a língua portuguesa (Brasil), adaptação cultural e validação do *Parathyroid Assessment of Symptoms* (PAS) em pacientes com doença renal crônica e hiperparatireoidismo

**DOI:** 10.1590/2175-8239-JBN-2022-0059pt

**Authors:** Rafael Costa e Campos, Rayssa Ruszkowski do Amaral, Marta Ribeiro Hentschke, Marcelo Garcia Toneto, Carlos Eduardo Poli-de-Figueiredo

**Affiliations:** 1 Pontifícia Universidade Católica do Rio Grande do Sul, Escola de Medicina, Porto Alegre, RS, Brasil.

**Keywords:** Hiperparatireoidismo, Qualidade de Vida, Insuficiência Renal Crônica, Reprodutibilidade dos Testes, Estudos de Validação

## Abstract

**Introdução:**

A doença renal crônica (DRC) está relacionada à elevada morbimortalidade e perda de qualidade de vida. Igualmente, o hiperparatireoidismo está associado à perda progressiva da função renal, com níveis aumentados de fosfato e diminuição de cálcio, que induzem secreção do paratormônio.

**Objetivos:**

Traduzir para língua portuguesa (Brasil), adaptar culturalmente e validar o questionário *Parathyroid Symptoms Assessment* (PAS), seguindo critérios de confiabilidade e validade em pacientes com DRC e hiperparatireoidismo.

**Métodos:**

Estudo metodológico e transversal, realizado no Hospital São Lucas/PUCRS, Porto Alegre, Brasil. O processo de validação do questionário PAS seguiu protocolos de estudos anteriores. Após tradução para o português, aplicou-se a 100 pacientes com hiperparatireoidismo secundário (HPTS) e terciário ou persistente (HPTT). Para dados de validação do PAS, os pacientes responderam ao questionário *Short Form Health 36* (SF-36). Critérios de confiabilidade foram avaliados usando coeficiente de correlação intraclasse (CCI) e alfa de Cronbach (α-C). A validade foi avaliada pelo coeficiente de correlação de Spearman entre valores de PAS e SF-36.

**Resultados:**

A idade média dos participantes foi 55,6 ± 15,6 anos, 61% eram homens e 68% foram diagnosticados com HPTS. Entre 100 pacientes, 53% realizaram reteste de PAS (CCI = 0,83). A confiabilidade interna por α-C foi 0,86. Observaram-se correlações negativas entre questões do PAS e os domínios físico e mental do SF-36, que variaram de 0,3 a 0,7.

**Conclusão:**

A versão brasileira do questionário PAS mostrou-se válida e confiável. O questionário PAS pode ser usado para avaliar qualidade de vida em pacientes brasileiros com hiperparatireoidismo que falam português.

## Introdução

Estima-se que a doença renal crônica (DRC) possa afetar de 8 a 16% da população mundial. As principais causas de DRC são doenças com elevada prevalência e incidência, como o diabetes mellitus (DM) e a hipertensão arterial (HA)^1^. Pessoas com DRC estágio 5 (taxa de filtração glomerular (TFG) <15 mL/min/1,73m^2^) apresentam doença renal em estágio terminal e necessitam de terapia renal substitutiva por diálise ou transplante renal. Com a progressão da insuficiência renal e, geralmente com uma TFG ≤ 60ml/min/1,73m^2^, ocorrem alterações nas concentrações séricas do cálcio, fósforo, vitamina D e paratormônio (PTH), que estão associadas a calcificações vasculares e de partes moles, e alterações na remodelação óssea. Essa síndrome sistêmica é denominada distúrbio mineral e ósseo da doença renal crônica (DMO-DRC)^2^. As alterações metabólicas como hipocalcemia, hiperfosfatemia, deficiência de vitamina D vão estimular o aumento da secreção do PTH, estabelecendo o hiperparatireoidismo secundário (HPTS).

No Brasil, em torno de 50% dos pacientes em diálise são portadores de HPTS e geralmente apresentam sintomatologia importante como dores ósseas, articulares, mialgia, fraqueza muscular, prurido, irritabilidade e outros, que diminuem a qualidade de vida desses pacientes^3^. Com a evolução do HPTS, além das complicações citadas, os pacientes apresentam uma redução da densidade mineral óssea, alterações da microarquitetura óssea, que aumentam o risco de fraturas patológicas e de mortalidade. Pacientes que apresentam um HPTS grave, não responsivo ao tratamento clínico, são encaminhados à paratireoidectomia (PTx).

A avaliação de sinais, sintomas e qualidade de vida por meio de escalas ou questionários tem atraído o interesse dos profissionais de saúde, levando a inúmeras publicações nos últimos anos. Esses instrumentos têm o objetivo de avaliar os aspectos físicos, mentais e sociais do paciente e expressá-los como variáveis quantitativas.

O questionário “*Parathyroid Assessment of Symptoms*” (PAS) – é uma ferramenta desenvolvida por Pasieka e Parsons^4^, que avalia sintomas específicos do hiperparatireoidismo. Inicialmente, o questionário foi aplicado apenas em pacientes com hiperparatireoidismo primário (HPTP), pré e pós PTx. Posteriormente, foi validado também para HPTS e para hiperparatireoidismo persistente ou terciário (HPTT), que ocorre após o transplante renal. Esse questionário é utilizado para confirmar o diagnóstico da doença, avaliar a gravidade dos sintomas e comparar seus valores antes e após a PTx, verificando o impacto do sucesso do procedimento cirúrgico nesses sintomas. Anteriormente, instrumentos genéricos, como o *Short Form Health 36* (SF-36), eram usados para medir a qualidade de vida em pacientes com hiperparatireoidismo^5^.

Assim, devido à importância clínica do PAS, este estudo teve como objetivo traduzir, adaptar culturalmente e validar para a língua portuguesa (Brasil) o questionário PAS, seguindo critérios de confiabilidade e validade em pacientes com DRC e hiperparatireoidismo.

## Métodos

### Tradução e Adaptação Cultural

Inicialmente, a pesquisadora Pasieka JL, responsável pelo desenvolvimento do questionário PAS original, foi contatada a fim de obter permissão para traduzir o PAS para a língua portuguesa^6^. A tradução e a adaptação cultural seguiram diretrizes estabelecidas na literatura^7, 8^. O processo teve início com a tradução do PAS do idioma original (inglês) para o idioma alvo (português) por dois tradutores independentes cujo idioma nativo é a língua portuguesa. As duas traduções foram analisadas e uma primeira versão foi produzida. Em seguida, realizou-se uma retrotradução, na qual a primeira versão em português foi retrotraduzida para o idioma original por um terceiro tradutor profissional cujo idioma nativo também é o português. A segunda versão (inglês) foi analisada e comparada com a primeira versão (português) por cinco especialistas da área e pelo autor do questionário original. Para a adaptação cultural, a análise foi realizada por um grupo de especialistas da área selecionados pelos autores. O pré-teste foi aplicado em cinco pacientes: dois com HPTP, dois com HPTS e um com HPTT^9^.

### Desenho do Estudo e Amostra

O estudo caracterizou-se como metodológico na fase de tradução e adaptação cultural e como transversal na fase de validação. Para a fase de validação foram incluídos 100 pacientes, homens e mulheres, com idade igual ou superior a 18 anos, com diagnóstico de DRC e HPTS ou pacientes transplantados renais com HPTT, que foram submetidos a acompanhamento e tratamento na Unidade de Nefrologia e Diálise (Hospital São Lucas (HSL)/PUCRS, Porto Alegre, Brasil). Todos os pacientes eram fluentes na língua portuguesa. Ambos os questionários foram aplicados após a obtenção de um consentimento informado por escrito assinado pelo participante.

O estudo foi aprovado pelo Comitê Institucional de Ética e Pesquisa Científica (No. 2.292.273-CEP).

#### Definições operacionais de HPTS e HPTT

HPTS: Presença de PTH elevado (valor de referência (VR) 15 a 68,3 pg/mL), cálcio normal ou baixo (VR de cálcio total 8,4 a 10,2 mg/dL) em pacientes com DRC estágio 5.

HPTT: Presença de PTH elevado, cálcio normal ou elevado em pacientes transplantados renais.

Os VR utilizados foram aqueles recomendados pelo hospital onde o estudo foi realizado.

#### Critérios de exclusão

Pacientes com limitações físicas que afetaram a capacidade de responder ao questionário e pacientes cujo tratamento foi alterado ou que foram submetidos a cirurgia (paratireoidectomia e transplante renal) durante o período entre as aplicações do questionário.

#### Cálculo da amostra

O cálculo amostral seguiu a diretriz de 5 a 10 participantes por item do questionário (o questionário PAS possui 13 itens)^10^.

### Dados Demográficos, Clínicos e Bioquímicos

As variáveis demográficas (sexo, etnia), clínicas (idade, peso, altura, tipo e tempo de diálise, tempo desde o transplante renal) e laboratoriais (PTH, creatinina, cálcio séricoe e fosfatase alcalina) dos pacientes foram obtidas dos registros do HSL.

### Instrumentos de Medição

#### Avaliação da qualidade de vida

#### Avaliação de sintomas da paratireoide

O PAS é um questionário específico para pacientes com hiperparatireoidismo, composto por 13 questões (físicas, mentais e sociais). Cada item é pontuado de acordo com a resposta em uma escala visual analógica que varia de zero (sem sintomas) a 100 (sintomas extremos). A pontuação do PAS é calculada como a soma de todas as 13 respostas, com uma pontuação máxima de 1.300.

#### SF-36, short form health 36

O SF-36 é um instrumento genérico de qualidade de vida composto por 36 questões, em que o paciente responde a perguntas variadas com pontuações entre 1 ou 2, 1 a 3, 1 a 5 e 1 a 6. Os 36 itens estão divididos em 8 domínios: capacidade funcional, aspectos físicos, dor, estado geral de saúde, vitalidade, aspectos sociais, aspectos emocionais e saúde mental. Cada domínio gera uma pontuação entre zero (pior estado) e 100 (melhor estado). O instrumento SF-36 foi aplicado na população do estudo como padrão-ouro, para que a validade do critério de construção pudesse ser avaliada.

Os questionários PAS e SF-36 foram aplicados pela mesma pessoa a todos os pacientes.

### Confiabilidade e Validade

A confiabilidade do instrumento foi avaliada usando reprodutibilidade por correlação intraclasse (teste/reteste)^11, 12^ e a consistência interna por alfa de Cronbach (α-C)^13^. Ao calcular o coeficiente de correlação intraclasse (CCI) entre duas aplicações (teste/reteste), avaliou-se a capacidade do instrumento de fornecer resultados semelhantes em um determinado período de tempo. A validade convergente foi realizada por meio da avaliação da correlação entre os resultados obtidos no PAS e no SF-36.

### Testes Estatísticos

Os testes estatísticos foram realizados utilizando o programa RStudio (versão 1.0.143–2009–2016 RStudio^©^, Inc.). As variáveis quantitativas são apresentadas como média ± desvio padrão (DP) ou mediana e intervalo interquartil (IIQ), conforme apropriado. As variáveis categóricas são apresentadas em porcentagens. As questões do PAS e os domínios do SF-36 são relatados como mediana e IIQ, além dos valores mínimo e máximo.

Para a análise da confiabilidade do teste/reteste, foi utilizado o CCI, com valores entre 0 e 1. De acordo com a literatura, valores de CCI <0,5 indicam baixa confiabilidade, entre 0,5 e 0,75 confiabilidade moderada, 0,75 a 0,9 boa confiabilidade e > 0,9 indicam excelente confiabilidade^14^. Para uma boa confiabilidade interna, o coeficiente de α-C deve ser > 0,7^15,16^.

A correlação entre os resultados do PAS e do SF-36 foi testada com o coeficiente de correlação de Spearman^17^. A hipótese nula foi rejeitada quando *p* < 0,05.

## Resultados

### Dados Demográficos, Clínicos e Bioquímicos

Entre janeiro e outubro de 2018, foram recrutados para validação do PAS em português um total de 100 pacientes com diagnóstico de HPTS (68%) e HPTT (32%) (61 homens; idade entre 21 e 81 anos, média de 55,6 ± 15,1 anos). As entrevistas foram realizadas durante o atendimento ambulatorial. O índice de massa corporal (IMC) médio foi de 26,9 ± 6,0 kg/m^2^. De acordo com a OMS, a maioria dos pacientes apresentava sobrepeso (IMC ≥ 25 < 30)^18^.

A doença subjacente que levou à DRC em pacientes com HPTS e HPTT foi DM em 22 pacientes, hipertensão em 16, doença urológica em 7, outras doenças renais em 32 e causas indeterminadas em 23. Entre outras doenças renais subjacentes, observaram-se glomerulopatias em 11 pacientes, doença renal policística em 8 pacientes, doenças autoimunes em 5, tumor e agenesia em dois pacientes cada e pielonefrite, Alport, tuberculose e nefropatia isquêmica em um paciente cada.

Entre os participantes diagnosticados com HPTS, 44 (64,7%) foram submetidos à hemodiálise como terapia renal substitutiva, 10 (14,7%) à diálise peritoneal e 6 (8,82%) realizaram ambas as terapias, nunca simultaneamente, antes do período do estudo. Oito pacientes (11,76%) ainda não haviam iniciado a terapia de diálise. A mediana do tempo desde o início da diálise até o dia da entrevista foi de 1,8 anos [IIQ 0,9–3,3]. Transplante renal prévio foi encontrado em 17 (25%) pacientes, que posteriormente evoluíram para perda da função do enxerto.

Entre os participantes com diagnóstico de HPTT, o tempo mediano pós-transplante até o dia da entrevista foi de 4,2 anos [IIQ 2,5–5,7]. Com relação ao tipo de doador, 31 eram provenientes de doadores falecidos (97,9%). O tempo mediano até a diálise, antes do transplante, foi de 3,9 anos [IIQ 2–5,8]. Quanto à terapia renal substitutiva, 21 (65,6%) estavam em hemodiálise, 4 (12,5%) em diálise peritoneal e 6 (18,7%) em ambas as terapias, nunca simultaneamente, antes do período do estudo. Entre os pacientes transplantados com HPTT, 50% apresentaram HPTS antes do transplante (durante o período de diálise). As características demográficas e clínicas são demonstradas na Tabela 1.

**Tabela 1:** Características demográficas e clínicas

Dados (n = 100)	
Sexo	
Masculino	61%
Idade (anos)	55,6 (±15,1)^1^
Peso (quilogramas)	74,87 (±16,7)^1^
Altura (metros)	1,67 (±0,1)^1^
IMC (kg/m^2^)	26,94 (±6,0)^1^
Cor da pele	
Branca	50%
Não-branca	42%
Sem resposta	8%
Hiperparatireoidismo	
Secundário	68%
Terciário	32%
Tempo desde o início da diálise até a entrevista (anos)	1,8 [0,9–3,3]^2^
Tempo pós-transplante até a entrevista (anos)	4,2 [2,5–5,7]^2^

IMC, índice de massa corporal.

1 Média (±desvio padrão);

2 Mediana [intervalo interquartil].

Os resultados dos testes bioquímicos, incluindo a taxa de filtração glomerular (TFG, utilizando conceitos da equação “*Chronic Kidney Disease Epidemiology Collaboration*” (CKD-EPI))^19^, são mostrados na Tabela 2.

**Tabela 2:** Exames laboratoriais e taxa de filtração glomerular (TFG)

Variável	Mediana [IIQ]
Creatinina (mg/mL)	
HPTS	8,8 [6,23–11,45]
HPTT	1,79 [1,55–3,02]
HPTS + HPTT	6,29 [3,23–9,57]
TFG estimada (mL/min/1,73m^2^)	
HPTS	5,55 [4,38–6,48]
HPTT	44,3 [19,57–55,3]
HPTS + HPTT	7,9 [4,87–17,03]
PTH (pg/mL)	
HPTS	504,3 [274,4–884]
HPTT	242,25 [143,8–339,1]
HPTS + HPTT	425 [233,7–733,2]
Cálcio sérico (mg/dL)	
HPTS	8,95 [8,58–9,5]
HPTT	10,1 [9,3–11,03]
HPTS + HPTT	9,2 [8,7–9,83]
Fosfatase alcalina (U/L)	
HPTS	124 [85–164]
HPTT	114 [83,5–195]
HPTS + HPTT	123 [85–165,5]

PTH: Paratormônio; [IIQ]: intervalo interquartil; HPTS: Hiperparatireoidismo secundário; HPTT: Hiperparatireoidismo terciário.

### Instrumentos de Medição

Os questionários PAS e SF-36 foram aplicados em 100 pacientes. A segunda aplicação do PAS foi realizada em 53 desses pacientes, com uma mediana de 16 dias entre as duas aplicações. Dos 53 pacientes, 45 (84,1%) apresentaram HPTS e 8 (15,1%) apresentaram HPTT. Não foi feita nenhuma aplicação secundária do SF-36.

O CCI entre as duas aplicações (teste/reteste) foi de 0,83. Ao incluir somente aplicações com intervalo ≤16 dias (32 pacientes), o CCI foi de 0,84. O α-C para confiabilidade interna foi de 0,86 em toda a amostra (HPTS = 0,86 e HPTT = 0,88). O valor mínimo foi de 0,82 e o máximo foi de 0,9 para as 13 questões do PAS. A confiabilidade e os dados do PAS são mostrados na Tabela 3.

**Tabela 3:** Valores do *parathyroid assessment symptoms* (PAS) e testes de confiabilidade

Variável	Mediana [IIQ]
PAS	
HPTS	340 [237,5–512,5]
HPTT	290 [230–515]
HPTS + HPTT	332,5 [230–512,5]
Intervalo entre aplicações do PAS (dias)	16 [14–21]
Coeficiente de correlação intraclasse^1^	0,83
Alfa de Cronbach	
HPTS	0,86
HPTT	0,88
HPTS + HPTT	0,86

[IIQ]: intervalo interquartil.

Valores de PAS variam de 0 a 1300.

1 Calculado em 53 pacientes que realizaram a segunda entrevista do PAS.

HPTS: Hiperparatireoidismo secundário; HPTT: Hiperparatireoidismo terciário.

Os valores dos itens do PAS foram (intervalo de 0 a 100): dor nos ossos 10 [0–42,5]; sentir-se cansado facilmente 35 [8,75–72,50]; alterações de humor 30 [0–50]; sentir-se deprimido 30 [0–60]; dor no abdômen 0 [0–30]; sensação de fraqueza 30 [10–50]; sensação de irritabilidade 30 [0–50]; dor nas articulações 30 [0–60]; esquecimento 30 [8,75–50]; dificuldade para levantar-se de uma cadeira ou sair do carro 10 [0–40]; dores de cabeça 0 [0-30]; prurido cutâneo 17,5 [0–50]; sede 45 [20–72,5].

Os valores da escala SF-36 foram (intervalo de 0 a 100): capacidade funcional (10 itens) 55 [35–81,25]; função-física (7 itens) 50 [0–100]; dor no corpo (2 itens) 61 [41–74]; estado geral de saúde (5 itens) 47 [35–67]; vitalidade (4 itens) 60 [43,75–70]; aspectos sociais (1 item) 75 [50–100]; função-emocional (5 itens) 66,67 [0–100] e saúde mental (1 item) 68 [52–84].

As correlações entre a primeira aplicação do PAS e o SF-36 estão descritas na Tabela 4. São apresentados os valores de correlação de Spearman e sua significância (*p*).

**Tabela 4:** Correlação de spearman entre os resultados do *parathyroid assessment symptoms* (PAS) e do short form health 36 (SF-36)

	*SF–36*							

*PAS*	CF	FF	Dor	EGS	VT	AS	FE	SM
*Dor nos ossos*	−0,33^1^	−0,33^1^	−0,47^1^	−0,23^2^	−0,35^1^	−0,32^2^	−0,08	−0,30^2^
*Sentir-se cansado facilmente*	−0,47^1^	−0,28^2^	−0,33^1^	−0,30^2^	**−0,51^1^**	−0,36^1^	−0,11	−0,41^1^
*Alterações de humor*	−0,15	−0,14	−0,19	−0,30^2^	−0,39^1^	−0,39^1^	−0,25^2^	**−0,61^1^**
*Sentir-se deprimido*	−0,23^2^	−0,13	−0,14	−0,36^1^	−0,34^1^	−0,28^2^	−0,29^2^	**−0,65^1^**
*Dor no abdômen*	−0,14	−0,15	−0,39^1^	−0,02	−0,13	−0,19	0,04	−0,18
*Sensação de fraqueza*	−0,44^1^	−0,33^1^	−0,36^1^	−0,39^1^	**−0,62^1^**	−0,40^1^	−0,19	−0,41^1^
*Sensação de irritabilidade*	−0,12	−0,01	−0,14	−0,19^2^	−0,32^2^	−0,26^2^	−0,14	−0,48^1^
*Dor nas articulações*	−0,36^1^	−0,31^1^	**−0,62^1^**	−0,29^2^	−0,27^2^	−0,33^1^	−0,11	−0,29^2^
*Esquecimento*	−0,07	−0,06	−0,06	−0,06	−0,17	−0,17	−0,03	−0,23^2^
*Dificuldade para levantar-se de uma cadeira ou sair do carro*	**−0,61^1^**	**−0,50^1^**	−0,42^1^	−0,34^1^	**−0,51^1^**	**−0,52^1^**	−0,20^2^	−0,39^1^
*Dores de cabeça*	−0,13	−0,23^2^	−0,27^2^	−0,16	−0,30^2^	−0,25^2^	−0,18	−0,31^1^
*Prurido cutâneo*	−0,34^1^	−0,41^1^	−0,37^1^	−0,41^1^	−0,49^1^	**−0,50^1^**	−0,17*	−0,39^1^
*Sede*	−0,37^1^	−0,30^2^	−0,32^2^	−0,20^2^	−0,33^1^	−0,25^2^	−0,15	−0,21^2^

CF: Capacidade funcional; FF: Função-física; EGS, Estado geral de saúde; VT: Vitalidade; AS: Aspectos sociais; FE: Função-emocional; SM: Saúde mental.

Magnitude da correlação. 0 a −0,1: trivial, −0,1 a −0,3: baixa, −0,3 a −0,5: moderada, −0,5 a −0,7: elevada, −0,7 a −0,9: muito elevada e −0,9 a −1: quase perfeita.

***Valores em destaque:*** correlação ≤ −0,5^1^. p < 0,001^2^ p < 0,05.

## Discussão

Neste estudo, o questionário PAS foi traduzido do inglês (idioma original do instrumento) para a língua portuguesa (Brasil), e a nova versão passou por uma adaptação transcultural (após análise de especialistas) e validação (comparando seus resultados com os do questionário SF-36).

O α-C obtido com a aplicação do questionário PAS à amostra do estudo foi elevado, comprovando a confiabilidade do novo instrumento. O CCI obtido por teste/reteste apresentou um resultado favorável, mesmo quando o intervalo entre as duas aplicações do PAS foi superior a 16 dias, atestando a confiabilidade do instrumento.

Para testar a reprodutibilidade de um instrumento através de teste/reteste, o tempo entre a primeira e a segunda aplicação não deve ser muito curto, para evitar que o paciente se lembre das respostas. Por outro lado, o tempo entre as aplicações também não deve ser muito longo, para que o entrevistador não adquira novas experiências que possam afetar os resultados. O ideal é que o teste/reteste seja realizado em um período de duas semanas^10,11^.

Na revisão da literatura, encontramos pelo menos três outros estudos que aplicaram o PAS em pacientes com HPTS^3,20,21^. Pasieka e Parsons^3^ avaliaram o impacto da paratireoidectomia nos sintomas em pacientes com HPTS e HPTT e estão em concordância com o presente estudo.

Com relação à aplicação do SF-36 em pacientes com diagnóstico de DRC e HPTS, estudos anteriores encontraram uma redução nos indicadores de qualidade de vida, principalmente nos itens relacionados à saúde física^22^, o que está em conformidade com nosso estudo^21,23, 24, 25^.

Os escores do PAS variam de zero (sem sintomas) a 100 (sintomas extremos) e o SF-36 varia de zero (pior estado) a 100 (melhor estado). Assim, ao estabelecer correlações entre o PAS e o SF-36, obtivemos valores negativos, mostrando uma correlação inversa entre ambos.

Estudos anteriores encontraram correlações significativas entre as questões do PAS e as pontuações do SF-36 em relação aos subgrupos físico e mental^20,26^. Esses estudos concluíram que o PAS (13 questões) pode fazer parte da rotina médica, uma vez que avalia mais especificamente os sintomas e a qualidade de vida de pacientes com hiperparatireoidismo.

No presente estudo, as correlações entre os dois questionários foram compatíveis com o conteúdo de cada questão. Por exemplo, as perguntas relacionadas a aspectos físicos no PAS estavam estreitamente relacionadas aos domínios físicos do SF-36, assim como as questões sobre bem-estar emocional estavam relacionadas aos domínios mentais.

Foram observadas correlações moderadas e elevadas em quase todas as análises entre o PAS e o SF-36. Esses resultados confirmam a consistência e a concordância de ambos os questionários.

Os resultados do PAS e do SF-36 sugerem que há uma perda de qualidade de vida em pacientes com hiperparatireoidismo, embora esse não tenha sido o objetivo principal do estudo.

Este estudo apresentou algumas limitações. Primeiramente, este foi um estudo transversal com uma amostra heterogênea que nos levou a considerar os valores de PTH, para definição de HPTS, segundo os estabelecidos pelo laboratório. Mesmo assim a mediana do PTH no HPTS foi de 504,3 [IIQ 274-884]. Adicionalmente, e apesar da sua relevância, os valores de fósforo séricos não foram apresentados, devido a escassez de dados nos prontuários. Além disso, tivemos dificuldades em realizar o reteste dentro de duas semanas, como ocorreu em estudos anteriores. No entanto, isso não parece ter influenciado o cálculo do CCI. A única questão do PAS que não apresentou correlação com nenhum domínio do SF-36 foi “Esquecimento”, provavelmente por não haver um item correspondente no SF-36. Por fim, nenhum dos itens do PAS e do SF-36 apresentou correlação muito elevada (*p* ≥ −0,7). A maioria das correlações foi moderada ou alta, o que parece ser suficiente para a validação do instrumento.

## Conclusão

A versão em língua portuguesa do PAS (Brasil) apresentou boa confiabilidade e validade e foi adaptada para avaliar a qualidade de vida de pacientes falantes de português com hiperparatireoidismo.

A aplicação clínica da presente versão do PAS antes e após uma intervenção médica ou cirúrgica fornecerá informações sobre a capacidade do teste de auxiliar diagnósticos, identificar sintomas e avaliar melhorias na qualidade de vida após os tratamentos.
